# Substitution in Amino Acid 70 of Hepatitis C Virus Core Protein Changes the Adipokine Profile via Toll-Like Receptor 2/4 Signaling

**DOI:** 10.1371/journal.pone.0131346

**Published:** 2015-06-29

**Authors:** Satoko Uraki, Masahiko Tameda, Kazushi Sugimoto, Katsuya Shiraki, Yoshiyuki Takei, Tsutomu Nobori, Masaaki Ito

**Affiliations:** 1 First Department of Internal Medicine, Mie University School of Medicine, Tsu, Japan; 2 Department of Molecular and Laboratory Medicine, Mie University School of Medicine, 2–174 Edobashi, Tsu, Mie, 514–8507, Japan; 3 Department of Gastroenterology and Hepatology, Mie University School of Medicine, Tsu, Japan; University of North Carolina School of Medicine, UNITED STATES

## Abstract

**Background & Aims:**

It has been suggested that amino acid (aa) substitution at position 70 from arginine (70R) to glutamine (70Q) in the genotype 1b hepatitis C virus (HCV) core protein is associated with insulin resistance and worse prognosis. However, the precise mechanism is still unclear. The aim of this study was to investigate the impact of the substitution at position 70 in HCV core protein on adipokine production by murine and human adipocytes.

**Methods:**

The influence of treatment with HCV core protein (70R or 70Q) on adipokine production by both 3T3-L1 and human adipocytes were examined with real-time PCR and enzyme-linked immunosorbent assay (ELISA), and triglyceride content was also analyzed. The effects of toll-like receptor (TLR)2/4 inhibition on IL-6 production by 3T3-L1 induced by HCV core protein were examined.

**Results:**

IL-6 production was significantly increased and adiponectin production was reduced without a change in triglyceride content by treatment with 70Q compared to 70R core protein in both murine and human adipocytes. IL-6 induction of 3T3-L1 cells treated by 70Q HCV core protein was significantly inhibited with anti-TLR2 antibody by 42%, and by TLR4 inhibitor by 40%.

**Conclusions:**

Our study suggests that extracellular HCV core protein with substitution at position 70 enhanced IL-6 production and reduced adiponectin production from visceral adipose tissue, which can cause insulin resistance, hepatic steatosis, and ultimately development of HCC.

## Introduction

Over 170 million people are infected with hepatitis C virus (HCV) worldwide. HCV is a major cause of liver damage, with virus-induced end-stage diseases such as liver cirrhosis and hepatocellular carcinoma (HCC) resulting in high rates of morbidity and mortality. Several factors are thought to influence outcomes after HCV infection. Viral factors include viral genotypes and viral load, and host factors include gender, age at infection, race [1, 2], single nucleotide polymorphisms upstream of *IL-28B* [3], and alcohol consumption.

In addition to factors described above that influence the disease outcome after HCV infection, it has been revealed that amino acid (aa) substitution at position 70 in the genotype 1b HCV core protein is also associated with worse prognosis. For example, several groups have reported that aa substitution at position 70 of core protein from Arg (70R) to Gln (70Q) is associated with non-virological response to interferon (IFN)-based therapy [4, 5]. Akuta et al. showed that genotype 1b-infected patients with 70Q had significantly higher Homeostasis model assessment-Insulin Resistance (HOMA-IR) scores compared to HCV-1b patients without substitution in this position, suggesting that the substitution has a close relationship to insulin resistance (IR) [6]. Moreover, it has also been suggested that 70Q is associated with a higher incidence of developing HCC [7, 8]. Thus, many clinical observations have suggested that HCV-1b infection with substitution at core 70 leads to resistance to IFN, IR, and hepatocarcinogenesis, yet the precise molecular mechanism for how substitution of a single aa can influence the disease outcome is still unclear. One hint was found by Funaoka et al. using a virus culture system. They showed that IFN signaling is suppressed in HCV-1b-infected cultured cells with core mutation (70Q/H), and that cellular expression of IL-6 and suppressor of cytokine signaling-3 (SOCS3) is significantly higher in core mutant-transfected cells than in wild-type-transfected cells [9].

Many clinical and experimental studies have suggested that HCV infection is relevant to metabolic disturbance. Moucari reported that IR is associated with genotype 1 and 4 HCV infection, and also accompanied by significant fibrosis [10]. By using a transgenic mouse model, Shintani et al. revealed that core protein is responsible for inducing hepatic IR by suppressing tyrosine phosphorylation of insulin receptor substrate (IRS)-1, leading to diabetes [11]. Liver steatosis is also a common feature of HCV infection, which lowers the likelihood of sustained viral response to IFN and accelerates liver fibrosis and HCC occurrence [12–14]. As one of the mechanisms of steatosis, Perlemuter suggested that HCV core protein inhibits both microsomal triglyceride (TG) transfer protein activity and secretion of very low-density lipoprotein (VLDL) [15].

There is accumulating evidence suggesting that visceral obesity is also related to HCV infection and worse disease outcome [16, 17]. Visceral adipose tissue is now regarded as an endocrine organ secreting several cytokines, called adipokines, that regulate energetic metabolism and influence IR, hepatic steotosis, and the development of HCC [18]. It has been revealed recently that toll-like receptors (TLRs) are expressed in human and murine adipocytes, and stimulation of these receptors affects the secretion of proinflammtory adipokines [19]. In addition, it has been reported HCV core protein is secreted into the bloodstream from infected hepatocytes [20], and that HCV core protein can trigger TLR2 signaling, inducing inflammatory activation [21], suggesting that HCV core protein can stimulate adipocytes via the TRL pathway.

Taken together, it is certain that single substitution to 70Q induced worsened prognosis, however, the precise mechanism is not clear yet and the influence of aa substitution of the HCV core protein on adipocytes has not been studied before. Therefore, in the current study, we investigated the impact of aa substitution at position 70 in HCV-1b core protein on adipokine production using murine and human adipocytes.

## Materials and Methods

### Human serum

Sera from patients infected with genotype 1b HCV were collected in Mie University Hospital between 2008 and 2011. HCV core sequences were analyzed in all patients. This study was approved by the Institutional Review Board of Mie University Hospital. Written informed consent was obtained from each patient included in the study. The study protocol conforms to the ethical guidelines of the 1975 Declaration of Helsinki as reflected in *a priori* approval by the institution's human research committee (Authorization number 859, 882).

### Recombinant HCV core protein

HCV RNAs were isolated from patients' sera infected with genotype 1b HCV using a Viral RNA Mini kit (Qiagen, Tokyo, Japan), and cDNA was synthesized by extension of random hexamers with PrimeScript reverse transcriptase (Takara Bio Inc., Otsu, Japan). After amino acid sequences of core protein in each virus was analyzed, a cDNA whose core protein amino acids were not substituted at either the 70th or 91st position (70R and 91L) was chosen to make 70R-type HCV core construct. After a PCR product covering the whole 70R-type HCV core protein was inserted into a TOPO TA cloning vector (Invitrogen, Carlsbad, CA), another HCV core construct which had a susbtitutin at 70th amino acid position (70Q) was synthesized by gene SOEing as previously described [22] and inserted to the TA cloning vector. The sense primer used for amplification of 70R HCV core was 5’-ATGAGCACAAATCCTAAACCTC-3’ and anti-sense primer was 5’-AGCGGAAGCTGGGATGGTCAAAC-3’. The sense primer for gene SOEing to make the 70Q construct was 5’-CAAGGCTCGCCAGCCCGAGGGTAG-3’ and anti-sense primer was 5’-AGGTCCTACCCTCGGGCTGGCGAG-3’. These constructs were inserted into a pGEX-6P (GE healthcare, Tokyo, Japan) and glutathione S-transferase (GST)-fusion recombinant vector. HCV core proteins (70R and 70Q) were synthesized using Takara Competent Cells BL21 (Takara Bio Inc.) according to the manufacturer’s instructions, and endotoxin was removed with a ProteoSpin Endotoxin Removal Micro Kit (Norgen, Thorold, Canada). Endotoxin level in each protein solution was measured by clinical laboratory testing company (SRL, Tokyo, Japan) and confirmed to be undetectable level.

### Cell lines and cell culture

Murine preadipocytes 3T3-L1 (JCRB9014) were purchased from the Health Science Research Resource Bank (Osaka, Japan). Cells were cultured in Dulbecco’s modified Eagle’s medium (DMEM) (Life Technologies, Tokyo, Japan) supplemented with 1% penicillin/streptomycin (Life Technologies) and 10% new born calf serum (NBCS) (Life Technologies) in a humidified atmosphere containing 5% CO_2_ at 37°C. For differentiation, two days after reaching confluence, media were changed for two days into DMEM with 10% fetal bovine serum (FBS) (Life Technologies) containing 5 μg/ml of insulin, 0.5 mmol/l of 1-methyl-3-isobutyl-xanthine, and 1 μmol/l of dexamethasone. Cells were further cultured with 10% NBCS-supplemented DMEM containing 5 μg/ml of insulin to differentiate them into adipocytes. On day 10 after induction, recombinant HCV protein or control GST were added to the culture medium (final concentration 2 pmol/l), and total RNA was extracted from cells for real-time PCR (RT-PCR) after 24 h, and culture media were harvested after 48 h for enzyme-linked immunosorbent assay (ELISA).

Primary human preadipocytes were purchased from Lonza Walkersville (Walkersville, MD) and differentiated into adipocytes according to manufacturer’s protocol. Briefly, cells were seeded at 20,000 cells/well of a 48-well cell culture plate and maintained in 250 μl of Preadipocyte Growth Medium-2 (PGM-2) (Lonza) until confluent. To induce the preadipocytes to differentiate into adipocytes, media were changed to Adipocyte Differentiation Medium (Lonza) containing insulin, dexamethasone, indomethacin, and isobutyl-methylxanthine. On day 10 after induction, recombinant HCV protein or control GST were added to the culture medium (final concentration 2 pmol/l), and total RNA was extracted from cells for RT-PCR after 24 h, and culture media were harvested after 48 h for ELISA. All the experiments were performed in triplicated or 4 replicated samples and the experiments were repeated at least three times to confirm the data.

### RNA extraction and Quantitative RT PCR (qRT-PCR)

Total RNA was extracted from cultured cells with the RNeasy Mini Kit (Qiagen, Tokyo, Japan), cDNA was synthesized from 1 μg of total RNA by extension of oligo dT primers with PrimeScript reverse transcriptase (Takara Bio), and qRT-PCR was performed using the ABI prism 7300 Real-time PCR system (Applied Biosystems, Foster City, CA) with EagleTaq Master Mix Kits (Roche Molecular Systems, Branchburg, NJ). The primer sets are shown in Table 1. The expression levels of target genes from triplicate reactions were determined by normalization to β-actin in human or GAPDH in mouse according to the manufacturer’s instructions.

**Table 1:** Primer lists for real-time PCR.

organism	Gene		primer
mouse	IL-6	Forward	gctaccaaactggatataatcagga
		Reverse	ccaggtagctatggtactccagaa
	adiponectin	Forward	gaatgtggaccaggcctct
		Reverse	ccccatccccatacacct
	Leptin	Forward	tgttagccctgaatgctgaag
		Reverse	agccaaggtttcttccctct
	GAPDH	Forward	agcttgtcatcaacgggaag
		Reverse	tttgatgttagtggggtctcg
human	IL-6	Forward	gatgagtacaaaagtcctgatcca
		Reverse	ctgcagccactggttctgt
	TNF-α	Forward	cagcctcttctccttcctgat
		Reverse	gccagagggctgattagaga
	β-actin	Forward	aagtcccttgccatcctaaaa
		Reverse	atgctatcacctcccctgtg

IL-6: interleukin 6, GAPDH: Glyceraldehyde 3-phosphate dehydrogenase, TNF-α: Tumor necrosis factor-α

### ELISA

Concentrations of mouse IL-6 (eBioscience, San Diego, CA), adiponectin (R&D Systems, Minneapolis, USA), and leptin (BioVendor, Heidelberg, Germany) in culture media were analyzed with ELISA. The concentrations of human IL-6 (eBioscience), adiponectin, and leptin(R&D Systems) in culture medium were also assessed with ELISA. All measurements were performed according to the manufacturer’s instructions.

### Measurement of TG content in adipocytes

TG was extracted from 3T3-L1 cells with methanol and chloroform. TG concentrations were measured with a Triglyceride E-test (Wako Pure Chemical Industries, Osaka, Japan) according to the manufacturer’s instructions. TG contents were normalized by protein concentration measured with Protein Assay (Bio-Rad, Hercules, CA).

### Inhibition of TLR2/4

It has been reported that HCV core protein stimulates the TLR2 pathway and induces IL-6 and IL-8 production [21]. Moreover, TLR2 and TLR4 mRNA have been reported to be expressed in adipocytes [19], and our data also showed that 3T3-L1 cells expressed TLR2/4 mRNA (data not shown). To elucidate the mechanism by which HCV core proteins, especially 70Q, stimulate adipocytes to cause IL-6 secretion, we performed inhibition experiments for TLR2 or TLR4. Inhibition of TLR2 was performed as previously described [23]. Briefly, 3T3-L1 cells were cultured in a 96-well plate and induced for differentiation into adipocytes. On day 10 after induction, 30 μg/ml of antagonistic monoclonal antibody (mAb) (T2.5) to TLR2 (eBioscience, San Diego, CA) or control IgG were added to each culture media. Thirty minutes later, 50 ng/ml of Pam_3_CSK4 or 2 pmol/l of recombinant HCV core protein (70R or 70Q) were added to each well and treated for 24 h, and the concentration of IL-6 in the culture medium was determined with ELISA. Inhibition of TLR4 was also performed with inhibitory peptide, VIPER (IMGENEX, San Diego, CA) according to the manufacturer’s protocol. Briefly, on day 10 after induction into adipocytes in a 96-well plate, 3T3-L1 cells were co-incubated with 5 μM of VIPER or control peptide (IMGENEX) for 2 h, and then 10 ng/ml of lipopolysaccharide (LPS) from *E*. *coli* (IMGENEX) or 2 pmol/l of recombinant HCV core protein were added to each culture medium and. After 24 h treatment, the concentration of IL-6 in the culture medium was determined with ELISA. Experiments were performed in 4 replicates and repeated at least twice independently.

### Statistical analysis

Comparisons of gene expression and cytokine production were performed using the two-tailed Student’s *t*-test. P < 0.05 was considered statistically significant.

## Results

### Mouse IL-6 is overexpressed in 3T3-L1 cells treated with 70Q

**Fig 1:**
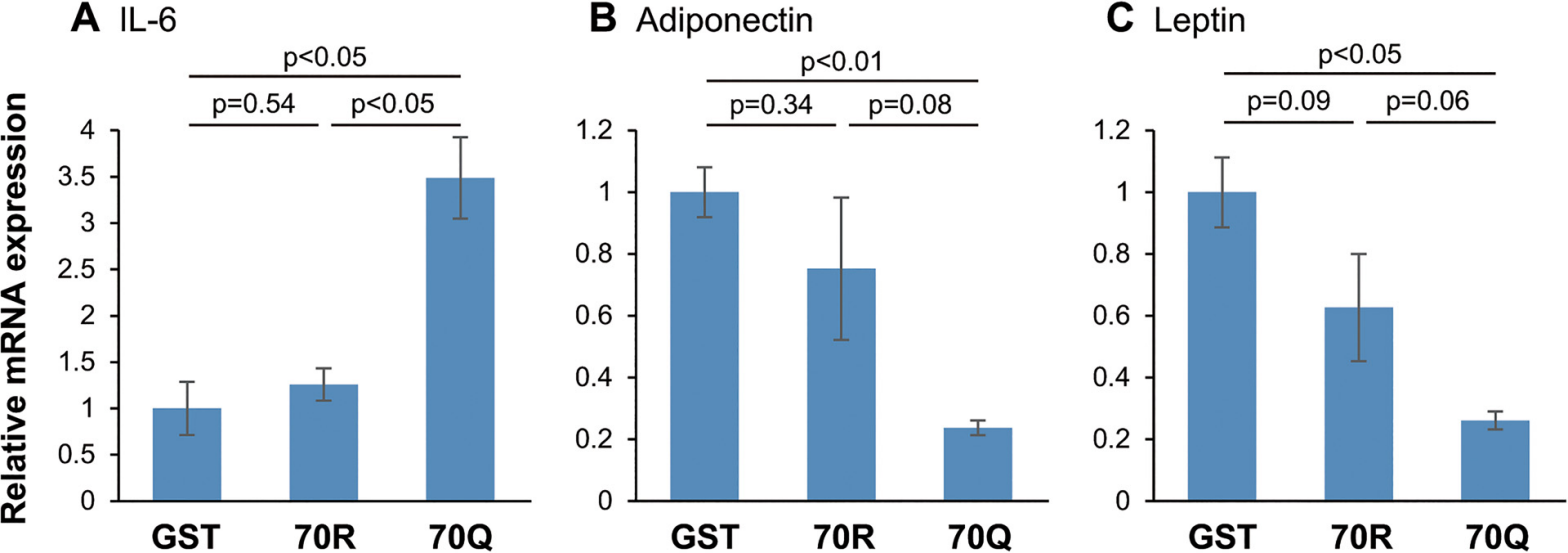
Changes in mRNA of adipokines in 3T3-L1 cells treated with HCV core protein. (A) Expression of IL-6 mRNA was significantly increased in 3T3-L1 cells treated with 2 pmol/l of 70Q for 24 h compared with 70R HCV core protein. The levels of (B) adiponectin and (C) leptin mRNA expression were reduced in 70Q cells compared to 70R, though they did not reach statistical significance.

**Fig 2:**
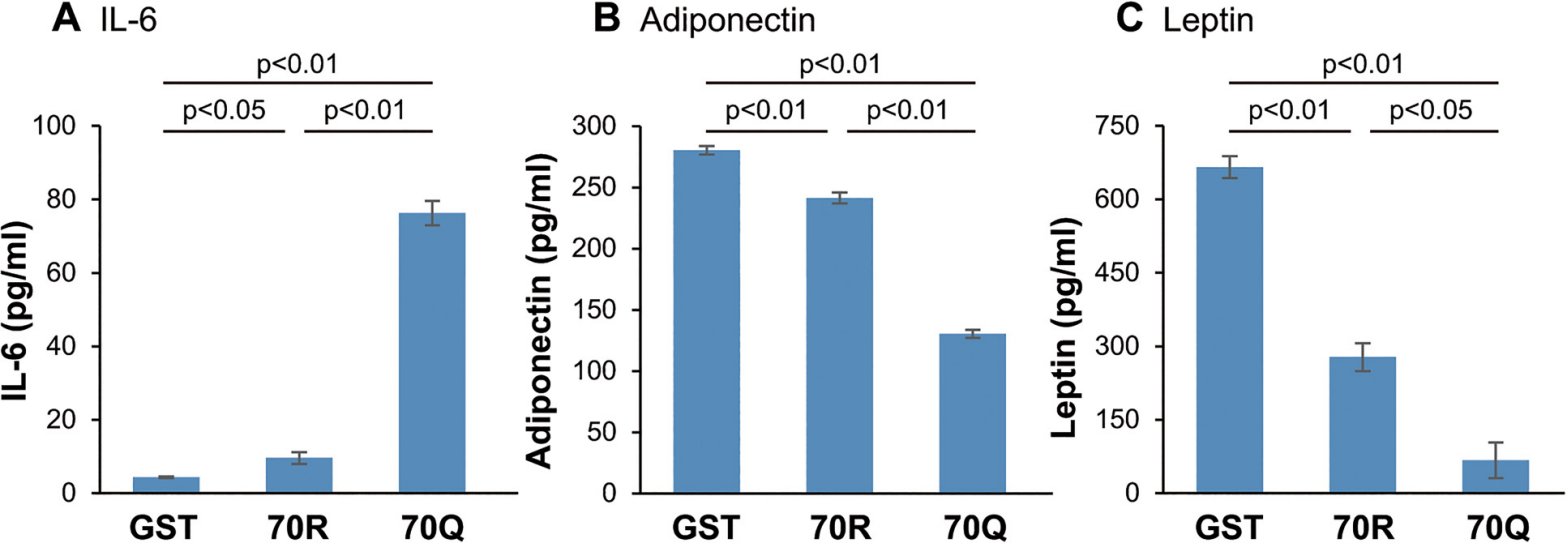
Changes in protein levels of adipokines in 3T3-L1 cells treated with HCV core protein. (A) The protein level of IL-6 was significantly increased, and the protein levels of (B) adiponectin and (C) leptin were significantly reduced in cells treated with 70Q for 48 h compared with 70R and GST protein. All data are expressed as the average ± SEM of three independent experiments. The levels of mRNA are shown as ratios relative to GST treated cells.

**Fig 3:**
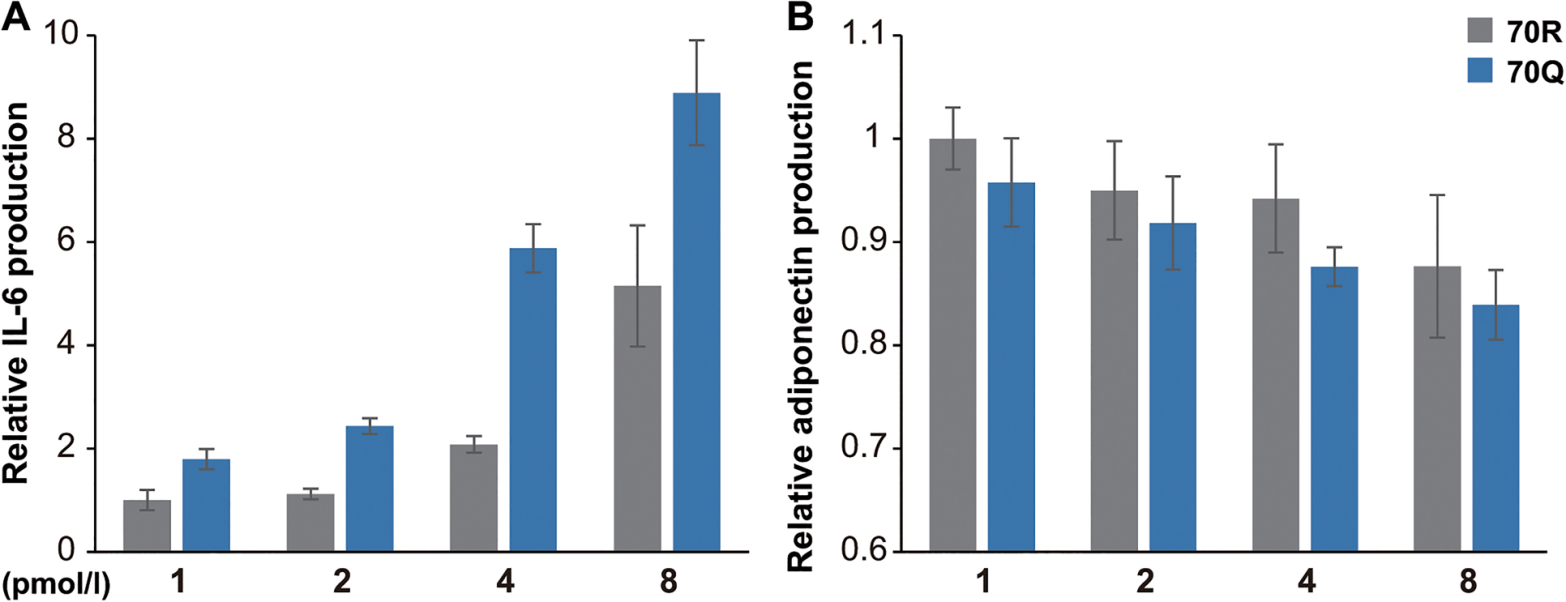
Changes in protein levels of IL-6 by different concentration of 70Q. (A) 70Q protein increased the production of IL-6 from 3T3-L1 cells in a dose-dependent manner. (B) 70Q protein reduced the production of adiponectin production from 3T3-L1 cells in a dose dependent manner. IL-6 production was higher and adiponectin production was lower in the cells treated with 70Q compared to 70R in each concentration.

First, we treated mouse 3T3-L1 cells, which differentiated into adipocytes, with HCV core recombinant protein. Since it has been reported that adipocytes secrete adipokines such as IL-6, TNF-α, adiponectin, and leptin, we examined mRNA expressions of these adipokines in 3T3-L1 cells treated with 2 pmol/l of GST, 70R, and 70Q protein by qRT-PCR. The level of IL-6 mRNA expression was significantly increased in 3T3-L1 cells treated with 70Q for 24 h compared with wild HCV core protein (Fig 1A). On the other hand, 70Q induced significant reduce in mRNA expressions of adiponectin and leptin compared to control (GST), though the differences between 70R and 70Q-treated cells did not reach statistical significance due to wide variation of data range (Fig 1B and 1C). There was no change in mRNA expression of other adipokine such as TNF-α, PAI-1 and resistin (data not shown). Next, we performed ELISA to measure the changes in protein levels of these adipokines. Similar to the changes in mRNA expression, the protein level of IL-6 was significantly increased in 70Q cells (Fig 2A), and the levels of adiponectin and leptin were significantly reduced in 70Q cells (Fig 2B and 2C). The level of TNF-α could be measured by neither RT-PCR nor ELISA because of its low expression levels. Moreover, 70Q HCV protein augmented the IL-6 production and reduced adiponectin production from 3T3-L1 cells in a dose-dependent manner (Fig 3A and 3B) and IL-6 production was higher and adiponectin production were lower in the cells treated with 70Q compared to 70R in each concentration.

### Human IL-6 was augmented in human adipocytes treated with 70Q

**Fig 4:**
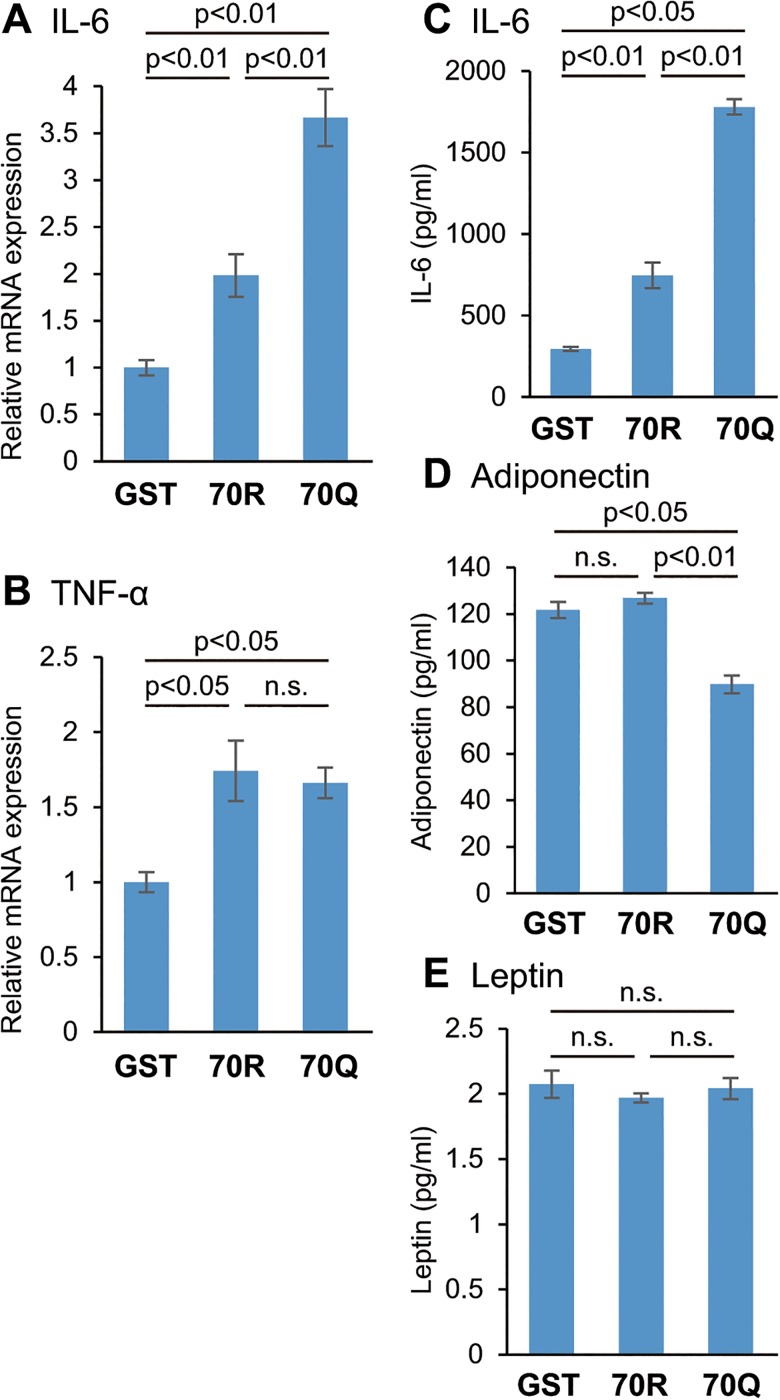
Changes in mRNA and protein levels of adipokines in human adipocytes treated with HCV core protein. (A) Expression of IL-6 mRNA was significantly increased in adipocytes treated with 2 pmol/l of 70Q for 24 h compared with wild HCV core protein. (B) TNF-α mRNA expression increased in HCV core-treated cells; however, there was no significant difference between cells treated with 70Q and 70R. (C) The protein level of IL-6 was significantly increased, and the protein levels of (D) adiponectin was significantly reduced in cells treated with 70Q for 48 h compared with 70R and GST protein. (E) However there was no change in leptin production. All data are expressed as the average ± SEM of five independent experiments. The levels of mRNA are shown as ratios relative to GST treated cells.

To investigate whether HCV core protein could also affect the expression levels of adipokines in human adipocytes, we treated human primary culture adipocytes with recombinant HCV core protein. Human IL-6 mRNA expression was significantly increased in adipocytes treated with 2 pmol/l of 70Q for 24 h compared with 70R HCV core protein (Fig 4A). TNF-α mRNA expression showed no significant difference between cells treated with 70Q and wild, though they were at detectable levels in these cells (Fig 4B). In addition, the protein level of IL-6 was significantly increased (Fig 4C), and the level of adiponectin was significantly reduced in cells treated with 70Q for 48 h (Fig 4D), though the production of leptin did not change between 70Q and 70R groups (Fig 4E)

### TG contents in 3T3-L1 cells

**Fig 5:**
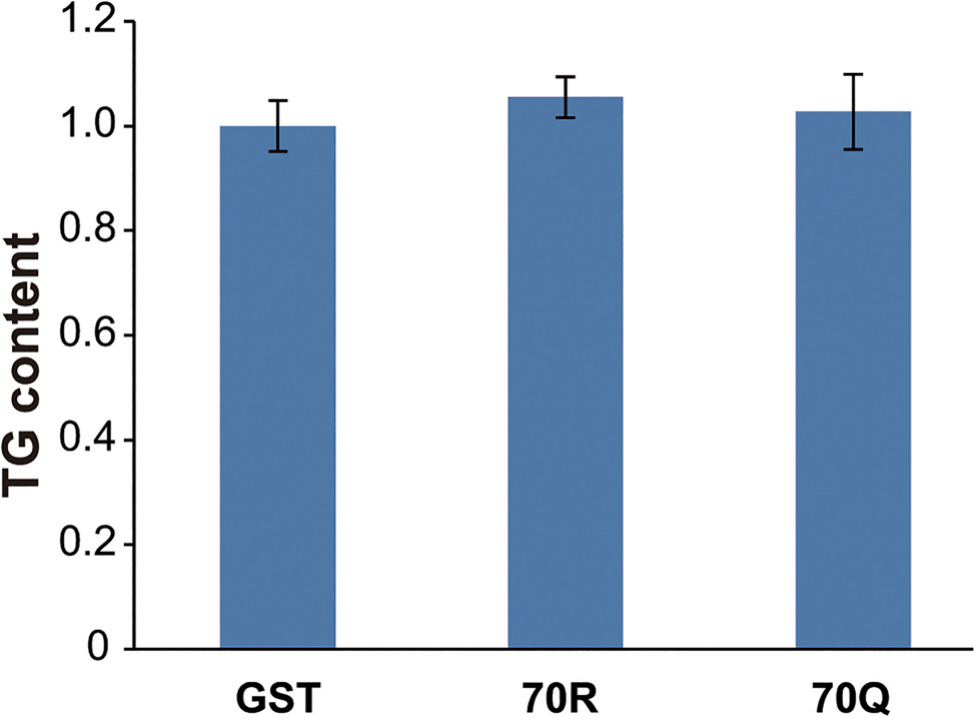
Changes in TG content. TG contents in 3T3-L1 cells were expressed relative to the control (GST-treated cells). There was no significant difference among GST, 70R, or 70Q core protein-treated cells.

TG ratios were calculated relative to GST-treated cells, and there was no significant difference among GST, 70R, or 70Q core protein-treated cells (Fig 5).

### IL-6 production in adipocytes by 70Q HCV core protein was reduced by inhibition of TLR2/4

**Fig 6:**
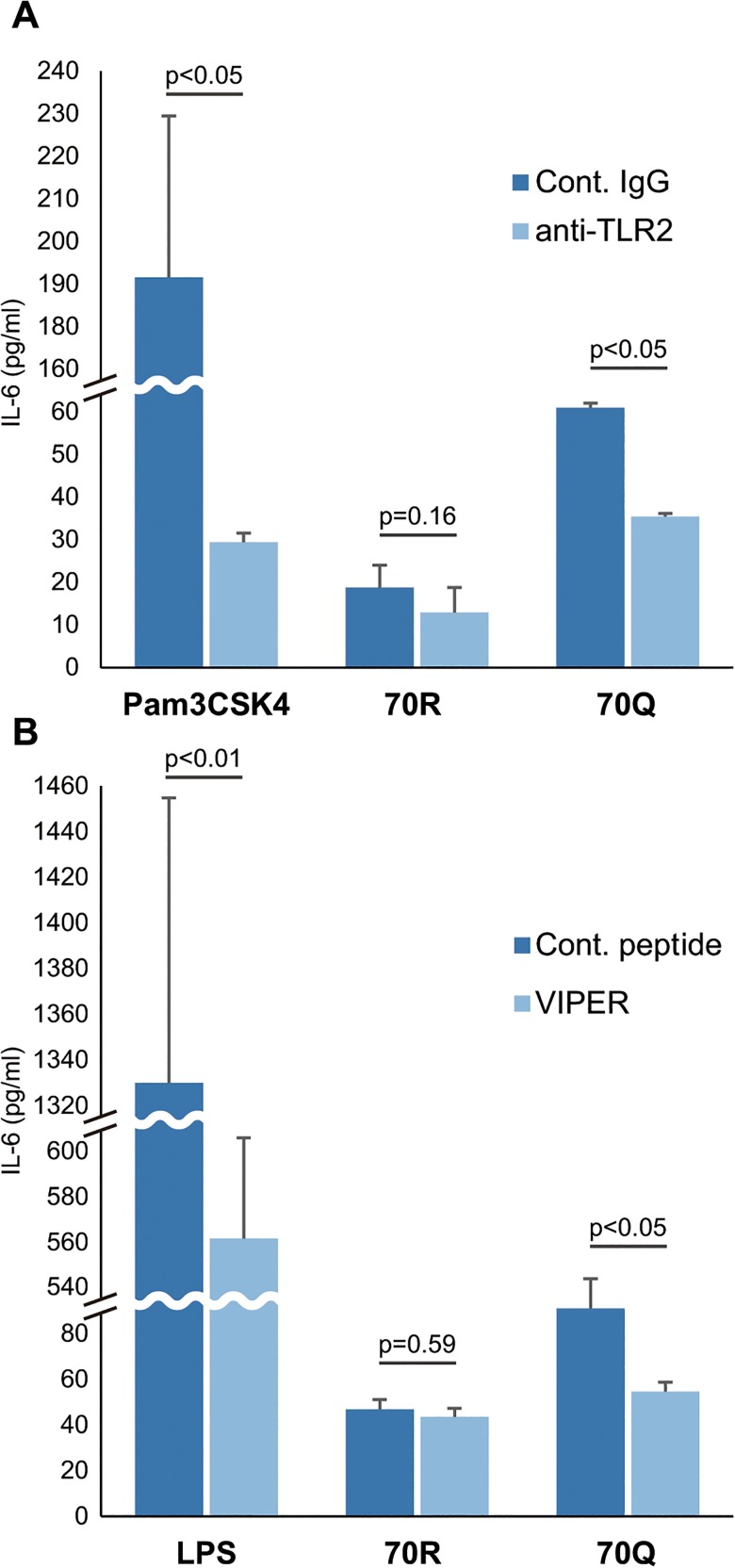
The influence of TLR2/4 inhibition on IL-6 secretion induced by HCV core protein. (A) 3T3-L1 cells stimulated by a TLR2 agonist, Pam3CSK4, induced IL-6 secretion, and it was inhibited by 85% with anti-TLR2 antibody. IL-6 induction of 3T3-L1 cells treated by HCV core protein was significantly inhibited with anti-TLR2 antibody by 42% in the 70Q group (p<0.05) and 31% in the 70R group, though it did not reach statistical significance, probably due to small sample size. (B) LPS induced IL-6 secretion, and VIPER inhibited it by 56%. IL-6 induction of 3T3-L1 cells treated by 70Q was significantly inhibited by VIPER, and the ratio of inhibition was 40% (p<0.05); however, it did not change IL-6 production with 70R HCV core protein.

3T3-L1 cells stimulated by a TLR2 agonist, Pam3CSK4, had remarkably induced IL-6 secretion, and it was inhibited by 85% with anti-TLR2 antibody. IL-6 induction of 3T3-L1 cells treated by HCV core protein was significantly inhibited with anti-TLR2 antibody by 42% in the 70Q group (p<0.05) and by 31% in the 70R group, though it did not reach statistical significance, probably due to the small number of samples (Fig 6A). LPS also induced marked IL-6 secretion, and VIPER, an antagonist of TLR4, inhibited it by 56%. IL-6 induction of 3T3-L1 cells treated by 70Q was also significantly inhibited by VIPER, and the ratio of inhibition was 40% (p<0.05); however, it did not change IL-6 production with 70R HCV core protein (Fig 6B). These results suggested that 70Q might induce IL-6 secretion via both TLR2 and TLR4, although production of IL-6 via TLR4 was not significant in cells treated with 70R HCV core protein.

## Discussion

In the current study, we demonstrated that HCV-1b extracellular core protein that has a substitution at position 70 significantly increased IL-6 production and decreased adiponectin production by both murine and human adipose cells compared to wild-type HCV-1b core protein, but the TG content did not change. Furthermore, this augmentation of IL-6 production by murine adipose cells was decreased by the addition of anti-TLR2 monoclonal antibody and TLR4 inhibitor.

An earlier study showed that HCV core transgenic mice had marked hepatic steatosis [24], suggesting that HCV core protein had some sort of causal effect on fat deposition in the hepatocytes. In chronic HCV-infected humans, hepatic steatosis is reported to be 2–3 fold more common compared to non HCV-infected subjects [25]. Although genotype 3 HCV infection is accompanied by more severe hepatic steatosis [26], it is also frequently observed in genotype-1 HCV infection [27, 28]. Moreover, hepatic steatosis in HCV infection leads to worse prognosis, increasing the incidence of HCC and decreasing the efficacy of IFN therapy [12–14]. Using a transgenic mouse model, Perlemuter et al. suggested that core protein has a direct effect on hepatic steatosis; it inhibits the assembly and secretion of VLDL causing TG accumulation in hepatocytes [15]. However, the precise mechanism for hepatic steatosis, especially in genotype-1 HCV infection, is still unclear, and the reason why genotype-1b infection with the 70Q substitution has poorer outcomes has not been fully studied.

In this study, we focused on the effects of HCV core protein on adipocytes, and found that treatment with 70Q HCV core protein significantly augmented IL-6 production by both murine and human adipocytes compared to 70R core protein, without changing the TG content. Adipose tissue is now thought to be the largest endocrine organ in the human body. It has been reported that adipocytes secrete several cytokines named adipokines, which include leptin, adiponectin, monocyte chemoattractant protein-1, etc. In addition, adipocytes can also secrete inflammatory cytokines such as TNF-α and IL-6, and as adipocytes grow bloated, they come to produce more inflammatory cytokines and less adiponectin [29]. These inflammatory cytokines produced by adipocytes play crucial roles in the pathogenesis of IR, hepatic steatosis, and HCC. Senn et al. reported that IL-6 induces up-regulation of SOCS-3 in human and murine hepatocytes and inhibits autophosphorylation of insulin receptor [30]. Furthermore, using mice in which *c-jun N-terminal protein kinase 1* (*Jnk1*) was ablated in adipose tissue, Sabio et al. suggested that adipose tissue secretes increased amounts of IL-6 in a JNK1-dependent manner and causes hepatic SOCS-3 production, leading to hepatic IR in mice fed with a high fat diet [31]. Their results also proved that IL-6 derived from adipose tissue can regulate distal metabolic effects in the liver. In human obesity, it has been suggested that a major source of IL-6 is adipose tissue. Indeed, Moschen et al. reported that IL-6 production is more than 100-fold higher in adipose tissue compared to liver [32]. As most IL-6 released from visceral adipose tissue is delivered to the liver at first via the portal vein, the liver is a key target organ. In hepatocytes, IL-6 also activates the janus-activated kinase-1 (JAK1)/signal transducers and activators of transcription 3 (STAT3) signaling pathway [33] and causes antiapoptotic responses and cell proliferation, ultimately leading to carcinogenesis [18]. The results of the current study clearly suggest that IL-6 production from visceral adipose tissue is augmented by 70Q HCV core protein, possibly causing worse prognosis.

On the other hand, our results showed that 70Q core protein induced a reduction in adiponectin production by both murine and human adipocytes. It has been suggested that adiponectin might function as an adipostat in regulating energy balance, and therefore its deficiency might contribute to the development of obesity and type 2 diabetes mellitus (DM). Adiponectin binds to at least two specific receptors named Adipo R1 and Adipo R2 [34]. Though Adipo R1 is widely expressed, most abundantly in skeletal muscle, Adipo R2 is predominantly expressed in the liver [35]. Adiponectin activates AMP-activated protein kinase (AMPK) and peroxisomal proliferator activated receptor (PPAR)-γ, causing increased fatty acid oxidation in the liver and skeletal muscle, whereas it causes decreased hepatic glucose production and decreased hepatic lipogenesis [36, 37]. In addition, Xu et al. reported that adiponectin suppresses hepatic production of TNF-α, which is one of the key cytokines that causes IR and steatosis in hepatocytes [38]. Moreover, adiponectin has been shown to up-regulate hepatic insulin receptor-2 (IRS-2) through STAT3 activation, thereby leading to insulin sensitization [39]. Levels of adiponectin in the blood are inversely correlated with fat mass and are decreased under conditions of obesity, IR, and type 2 DM, and patients with nonalcoholic steatohepatitis also have reduced serum adiponectin levels compared to control subjects [40]. Durante-Mangoni et al. and Zampio et al reported HCV-infected patient had reduced serum adiponectin levels [41, 42], and Simons et al. demonstrated that IL-6 reduces adiponectin secretion from human adipocytes [43], supporting our experimental results. Furthermore, our results showed the augmentation of IL-6 production and reduction of adiponectin production from adipocytes was dependent on HCV core protein dose, suggesting one reason for resistance to IFN therapy in patients with high viral loads.

Next, to clarify the mechanism of how HCV core protein with the substitution transmits signals to produce IL-6 in adipocytes without increasing the TG content, we investigated the influence of TLR2/4 inhibition on IL-6 secretion from adipocytes. Several studies have reported that TLRs are functionally expressed on adipocytes, and especially TLR2 and 4 play important roles in transmitting inflammatory signals in these cells [19, 44]. Besides its specific ligands, HCV core protein has also been reported to potentially stimulate TLR2 [21]. We found that inhibition of both TLR2 and 4 significantly reduced IL-6 secretions from adipocytes induced by 70Q core protein up to 42% and 40%, respectively. Interestingly, blocking TLR4 caused inhibition of IL-6 production induced by 70Q protein only; however, anti-TLR2 mAb inhibited both 70R protein and 70Q protein, suggesting that 70Q protein can stimulate both TLR2 and 4, while 70R protein can stimulate only TLR2. Thus, the results of the current study indicate that 70Q protein induces augmented IL-6 production by adipocytes at least partially via TLR2/4 signaling.

We acknowledge a limitation of this study is that our data were obtained from only cultured adipocytes; hence, further studies using animal models will be needed to investigate the effect of variant HCV core protein on production of these adipokines from visceral adipose tissue and to assess their levels in portal blood.

Tacke et al. demonstrated that HCV core protein activates STAT3 signaling in macrophages and dendritic cells via the autocrine IL-6 pathway [45], and Funaoka et al. showed that 70Q HCV core protein induces up-regulation of IL-6 and SOCS3 in hepatocytes [9], suggesting that it is one cause of worsened IFN resistance. In addition to their reports, our current study raises another possible scenario in which extracellular HCV core protein with the substitution (70Q) enhances IL-6 production and reduces adiponectin production from visceral adipose tissue, leading to insulin resistance, hepatic steatosis, and ultimately the development of HCC.
